# Tunable Ultrastrong Magnon–Magnon Coupling Approaching the Deep-Strong Regime in a van der Waals Antiferromagnet

**DOI:** 10.1021/acsnano.5c02576

**Published:** 2025-04-17

**Authors:** Charlie W. F. Freeman, Harry Youel, Adam K. Budniak, Zekun Xue, Henry De Libero, Thomas Thomson, Michel Bosman, Goki Eda, Hidekazu Kurebayashi, Murat Cubukcu

**Affiliations:** † London Centre for Nanotechnology, University College London, London WC1H 0AH, U.K.; ‡ National Physical Laboratory, Teddington TW11 0LW, U.K.; § Department of Physics, National University of Singapore, Singapore 117551, Singapore; ∥ Department of Materials Science & Engineering, National University of Singapore, Singapore 117581, Singapore; ⊥ Department of Computer Science, University of Manchester, Manchester M13 9PL, U.K.; # Institute for Materials Research and Engineering, Agency for Science, Technology and Research (A*STAR), Singapore 138634, Singapore; ¶ Department of Chemistry, National University of Singapore, Singapore 117543, Singapore; ∇ Centre for Advanced 2D Materials, National University of Singapore, Singapore 117542, Singapore; ○ Department of Electronic and Electrical Engineering, University College London, London WC1E 7JE, U.K.; ⧫ WPI-AIMR, Tohoku University, 2-1-1, Katahira, Sendai 980-8577, Japan

**Keywords:** spintronics, hybrid magnonics, 2D materials, antiferromagnets, spin dynamics

## Abstract

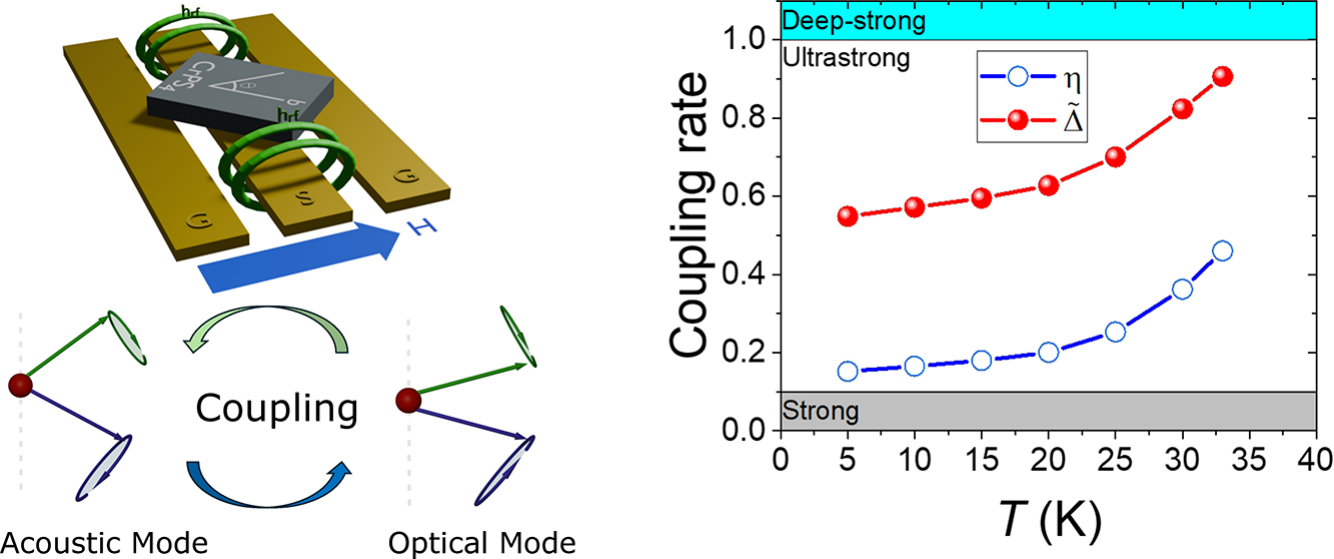

Antiferromagnetic (AFM) magnons in van der Waals (vdW) materials offer substantial potential for applications in magnonics and spintronics. In this study, we demonstrate ultrastrong magnon–magnon coupling in the GHz regime within a vdW AFM, achieving a maximum coupling rate of 0.91. Our investigation shows the tunability of coupling strength through temperature-dependent magnetic anisotropies. We compare coupling strength values derived from the gap size from the measured spectrum with those calculated directly through the coupling parameter and show that the gap size as a measure of coupling strength is limited for the ultrastrong coupling regime. Additionally, analytical calculations show the possibility to reach the deep-strong coupling regime by engineering the magnetic anisotropy. These findings highlight the potential of vdW AFMs as a model case to study magnetization dynamics in low-symmetry magnetic materials.

## Introduction

Within the expansive range of materials explored for spintronic applications, antiferromagnets (AFMs) have emerged as compelling candidates owing to their unique properties and potential to address challenges associated with traditional ferromagnetic (FM) devices.^1,2^ Characterized by antiparallel alignment of neighboring magnetic moments, net zero magnetization, and atomistic exchange coupling strength, AFMs offer inherent advantages such as insensitivity to external magnetic fields and ultrafast spin dynamics.^3^ However, the intricate nature of spin dynamics in AFMs presents both opportunities and challenges, underscoring the need for further investigation to fully harness their potential in spintronics.

Layered van der Waals (vdW) materials can be mechanically exfoliated down to monolayers relatively easily under controlled experimental conditions.^4^ The recent discovery of long-range magnetic order at the two-dimensional (2D) limit, which does not follow the Mermin-Wagner theorem^5^ due to finite magnetic anisotropies, has stimulated an intense exploration of the growing variety of magnetic 2D materials.^6,7^ Similar to synthetic antiferromagnets (SyAF),^8^ vdW AFMs such as CrCl_3_^9^ and CrPS_4_^10^ have a weak interlayer exchange coupling, which brings the typical terahertz (THz) dynamics of atomistic AFMs down to the gigahertz (GHz) regime. This characteristic makes them an ideal platform for studying a variety of spin dynamics phenomena using modern microwave techniques.

The interaction between magnons and other quasiparticles has garnered interest due to its fundamental importance in understanding complex quantum material interactions and its potential application in hybrid magnonics.^11,12^ Using the unique properties of magnons, these systems have shown significant promise in quantum information processing, storage, and sensing.^13,14^ Due to the inherently weak dipolar interaction between magnons and photons, it is often difficult to achieve ultrastrong magnon–photon coupling without extensive engineering and optimization.^15,16^ The coupling rate, typically defined as the ratio of the gap size to the bare excitation frequency, is used to quantify the rate of information transfer between coupled modes and defines the interaction regimes. When the coupling rate increases above 0.1, the ultrastrong coupling regime is realized.^17−19^ For light-matter systems in this regime, the rotating wave approximation does not hold and the gap size is no longer a good representation of the coupling strength. To account for this, more accurate models are adopted, such as the quantum Rabi model and Hopfield model.^20^ In ultrastrong coupling systems, the interaction Hamiltonian consists of both the corotating and counter-rotating terms. This permits exotic physical phenomena, such as nontrivial ground states and superradiant phase transitions.^21−23^ Recently, the interaction between magnons themselves has emerged as a new avenue for investigating coupling phenomena.^9,24−26^ Unlike magnon–photon coupling, a strong magnon–magnon coupling strength is in general expected due to their large spatial mode overlap.^21^

Quantifying the strength of magnon–magnon coupling remains a topic of considerable debate. The gap size is commonly used as a measure of magnon–magnon coupling strength, serving as a tool to define ultrastrong interactions in magnon–magnon systems.^21,26−29^ Recently, it has been shown that when intrinsic symmetry breaking induces the coupling, that an indirect gap opening occurs between the resonance modes with increased coupling strength than through extrinsic means.^30^ Additionally, a dc-field independent coupling strength parameter has been proposed for defining this coupling, which can be derived from material parameters in the case of SyAF with asymmetric magnetization between the layers.^30^

Ultrastrong magnon–magnon coupling has been experimentally observed in different material systems and explained by various symmetry breaking procedures from extrinsic origins (oblique dc fields) and intrinsic (Dzyaloshinskii–Moriya interaction and anisotropy).^21,24,27,28,31^ Using vdW AFMs, Li et al.^29^ demonstrated that the symmetry breaking due to magnetic anisotropy in CrPS_4_ enables ultrastrong magnon–magnon coupling that can be controlled by applying different magnetic fields with respect to the crystalline axes. Here, the gap size was used to identify the coupling rates leading to a maximum value of 0.31. Moreover, the deep-strong coupling regime has so far not been achieved experimentally in magnon–magnon interactions, with a recent study in SyAFs reaching values near to this.^21^

In this work, we demonstrate ultrastrong magnon–magnon coupling in the vdW AFM material CrPS_4_ in the GHz regime, achieving a coupling rate of 0.91. We show how the orthorhombic magnetic anisotropy lowers the symmetry of the magnetic system, thereby inducing magnon–magnon coupling between the two resonance modes. Furthermore, we investigate the temperature dependence of this coupling and report the magnetic parameters as a function of temperature. We demonstrate the tunability of coupling strength with temperature through controlling magnetic parameters, such as saturation magnetization and the magnetic anisotropy. We compare coupling rate values derived from gap size with those calculated directly from the intrinsic coupling parameter and discuss the difference between the two. Our model predicts a viable strategy to reach the deep-strong coupling regime by changing magnetic anisotropies.

## Results and Discussion

### Structure and Magnetism

**Figure 1:**
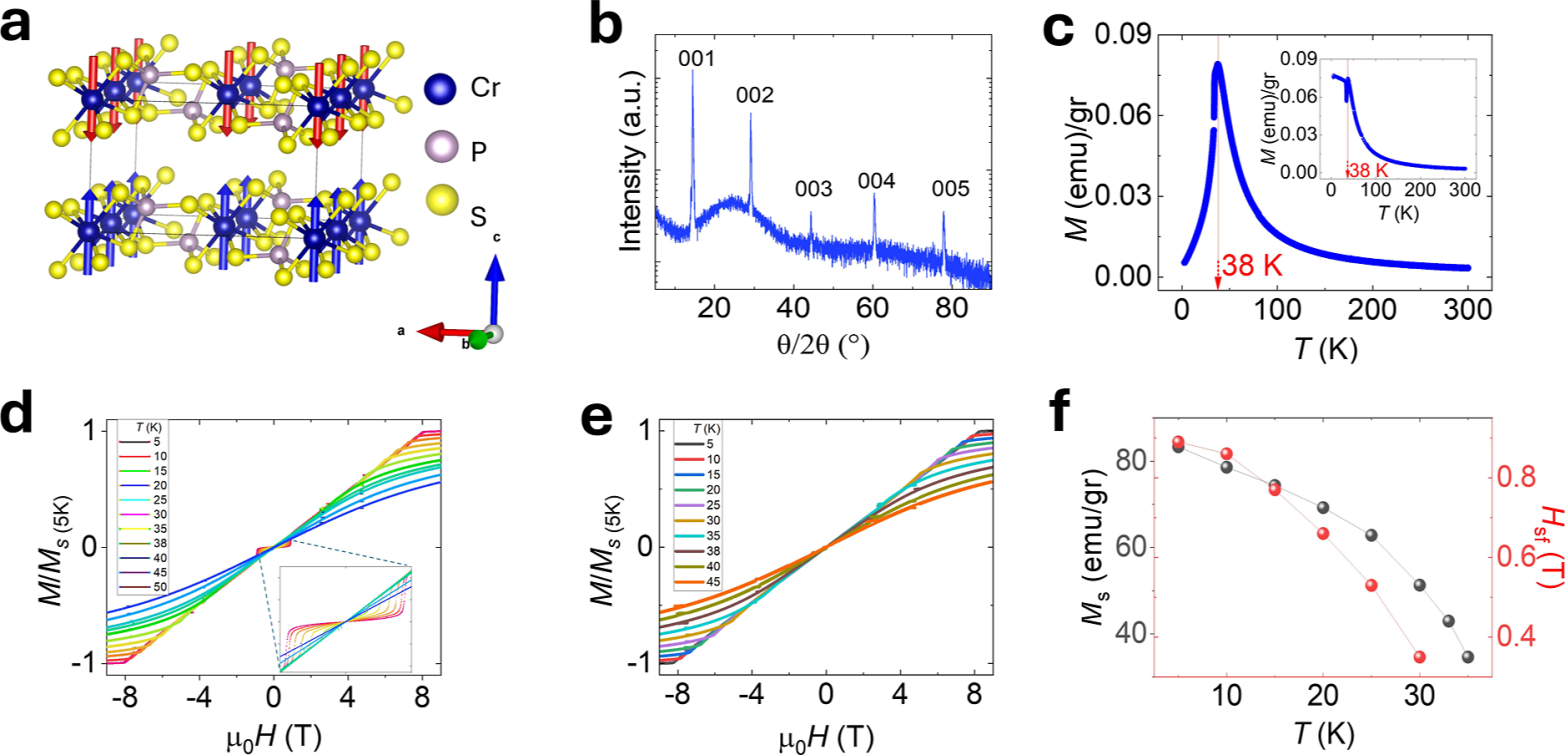
Structure and magnetism in CrPS_4_. (a) Schematic illustration of crystal and magnetic structures of CrPS_4_. The blue and red arrows indicate the orientation of magnetic moments. (b) X-ray diffraction of a single bulk CrPS_4_ crystal. (c) Zero-field-cooled magnetization (*M*) of a CrPS_4_ single crystal as a function of temperature (*T*), with a 10 mT magnetic field applied parallel to the crystallographic *c*-axis. The red line indicates *T*_N_. The inset illustrates the behavior under a magnetic field applied perpendicular to the *c*-axis. (d) Field-dependent normalized magnetization (*M*/(*M*_S(5K)_)) at various temperatures with the magnetic field (μ_0_*H*) oriented parallel to the *c*-axis. The inset provides a zoomed-in image around spin-flop transition (*H*_sf_). (e) Field-dependent normalized magnetization at various temperatures with the field-oriented perpendicular to the *c*-axis. (f) Saturation magnetization (*M*_s_) and spin-flop transition field (*H*_sf_) as a function of temperature (*T*).

CrPS_4_ is characterized by its semiconducting properties and an optically measured band gap of approximately 1.4 eV.^32,33^ The crystal structure exhibits noncentrosymmetricity with monoclinic anisotropy, belonging to space group C_2_, with crystal axes of lengths *a* = 10.871 Å, *b* = 7.254 Å, *c* = 6.140 Å, and β = 91.88°.^34^ The crystal structure is illustrated in Figure 1a. In Figure 1b, we show X-ray diffraction of a single bulk CrPS_4_ crystal. The position of 00x peaks is in agreement with work by Louisy et al.,^33^ confirming the successful growth of high-quality CrPS_4_.

We present the magnetometry results obtained from our bulk crystal in two field orientations, *H*//*c* and *H*//*ab*, as functions of the applied magnetic field, *H*, and temperature, *T*. In Figure 1c, we depict the magnetization, *M*, versus *T* under a small applied field (10 mT) for *H*//*c*. A sharp peak at 38 K is observed followed by a steep decline, indicative of the Néel transition temperature, *T*_N_, consistent with that in the literature.^10^Figure 1d,e plots *M* versus *H* for the *H*//*c* and *H*//*ab*, respectively, at *T* ranging from 5 K to 50 K. For *T* above 10 K, a prominent nonsaturating background is observed. From these, we extract the saturation magnetization as a function of *T*. In the *H*//*c* orientation, a characteristic spin-flop transition is evident due to the out-of-plane magnetic easy axis of the material.^10^ The spin-flop transition is indicated by the plateau in the moment until reaching a critical field, *H*_sf_. Beyond *H*_sf_, a sharp increase in *M* is observed as the sublattices become canted toward the direction *H*. With increasing *H*, a linear rise in *M* is measured until saturation, as the angle between the sublattices and *H* decreases, ultimately saturating in the forced FM phase around 8 T at 5 K. The extracted values of *M*_s_ and *H*_sf_ are plotted as functions of temperature in Figure 1f, where both values decrease with increasing temperature.

### Tunable Ultrastrong Magnon–Magnon Coupling Approaching the Deep-Strong Regime

We focus on ferromagnetic resonance (FMR) measurements conducted below the *T*_N_ for two orientations: *H*⊥*c* with θ ≈ 90° and 45°, where θ is the angle between the *b* axis and *H*. The crystal axis of the bulk crystal is determined visually, considering the straight edge of the crystal as the crystal growth aligns with the preferred *b* axis.^10^ This method of determination differs from the preferential edges observed during exfoliation, which typically exhibit an angle of 33.75° away from the *a* axis.^32,35^

We now present a detailed study of the temperature dependence of the resonances measured in the in-plane orientation with respect to the crystal axis, *b*, at angles θ ≈ 90° and 45°. Measurements in the out-of-plane orientation are included in the Supporting Infomation (Figures S1 and S2).

To model the spin dynamics, we employ the coupled Landau-Liftshitz-Gilbert (LLG) equation for two macrospin sublattices coupled through an interlayer exchange interaction, with zero damping for simplicity. Starting with Keffer’s free-energy equation for orthorhombic anisotropy and neglecting the terms dependent upon the relative positions of the sublattice moments^36^
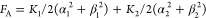
1where *F*_A_ is the anisotropy component free energy, *K*_1_ and *K*_2_ are the anisotropy constants related to the *a* and *b* axis, respectively, and α_1_, α_2_, β_1_, and β_2_ are the cosines of the unit magnetization for the two sublattices. The LLG equation can then be written as follows
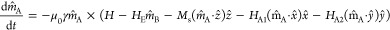

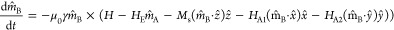
2where *m*_A_ and m_B_ are the moments of the two sublattices, respectively, γ is the gyromagnetic ratio, and *H*_E_ denotes the interlayer exchange field. The anisotropy fields, *H*_A1_ and *H*_A2_, are defined as *K*_1_/μ_0_*M*_s_ and *K*_2_/μ_0_*M*_s_, respectively. In this model, the crystal axes *a*, *b*, and *c* are aligned with the coordinate axes *x*, *y*, and *z*, respectively. The unit vectors of this coordinate system are denoted by *x̂*, *ŷ*, and *ẑ*.

**Figure 2:**
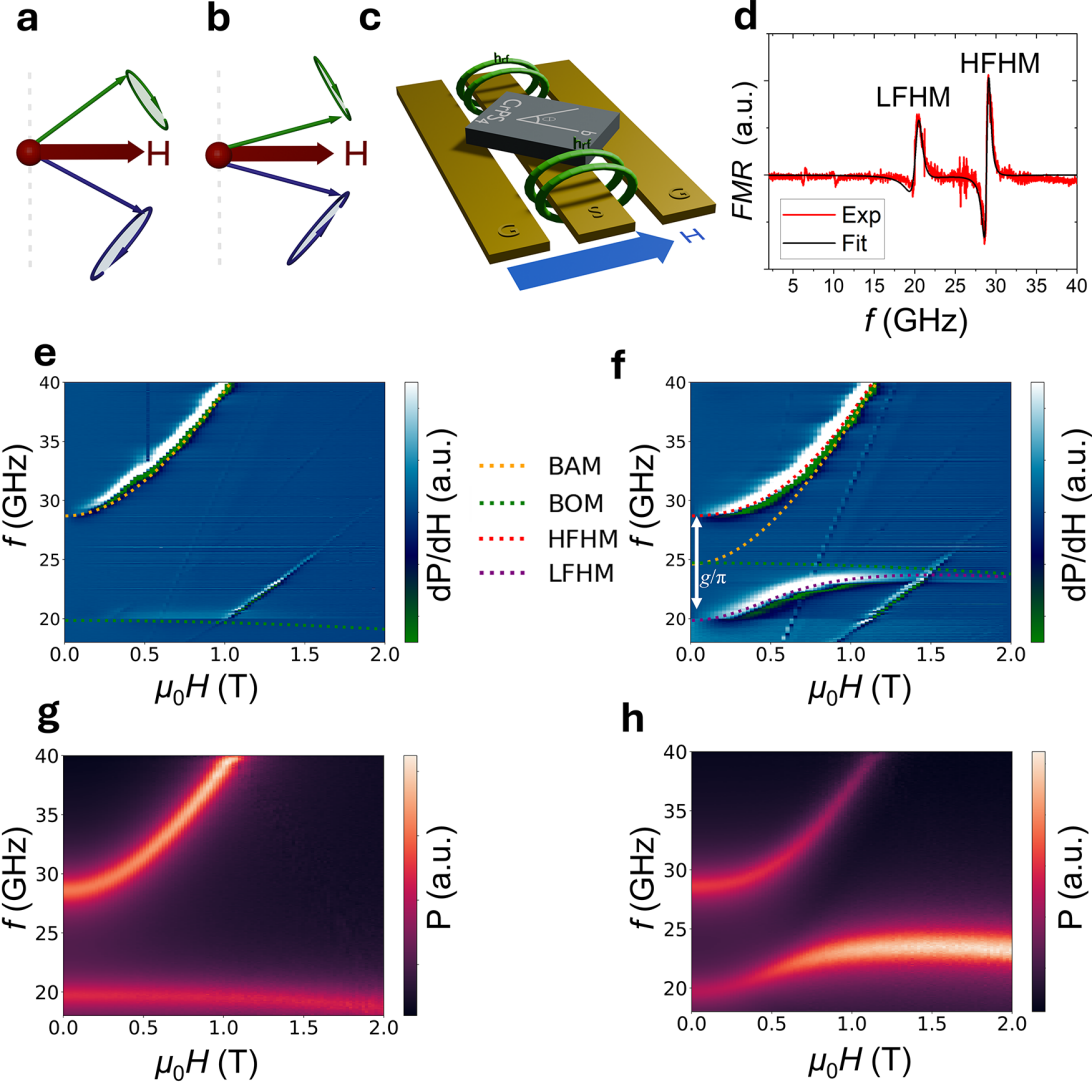
Spin dynamics in CrPS_4_ under an in-plane magnetic field. (a,b) Schematic illustration of the magnetic moments’ precession in the acoustic mode (in-phase precession) (a) and the optical mode (out-of-phase precession) (b). (c) Schematic of the configuration between coplanar waveguide (CPW), CrPS_4_ crystal, and external in-plane magnetic field (*H*). The angle between *H* and the *b* axis is shown by θ. (d) Typical ferromagnetic resonance (FMR) spectra (red line) obtained at 15 K and 200 mT and their derivated Lorentzian fitting (black line) by using eq S9 (see the Supporting Infomation). (e,f) 2D maps of the FMR spectra obtained at 15 K, with the magnetic field applied (μ_0_*H*) at θ = 90° (e) and θ = 45° (f). The fitted resonance modes are denoted as BAM, BOM, HFHM, and LFHM, representing the bare acoustic mode, bare optical mode, high-frequency hybrid mode, and low-frequency hybrid mode, respectively. The gap size *g*/π is depicted in (f) at 0 T where the uncoupled modes would intersect. (g,h) Simulated FMR spectra obtained with the parameters extracted from experimental data with μ_0_*H* at θ = 90° (g) and θ = 45° (h).

After Fourier transformation, the LLG equation can be treated as an 2 × 2 eigenvalue problem with the characteristic equation of
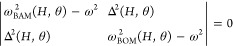
3where Δ denotes the intrinsic magnon–magnon coupling term and ω_BAM_ and ω_BOM_ are the bare acoustic and optical mode frequencies, respectively. These precession modes are depicted in Figure 2a and b, respectively.

Solutions to this reveal that when θ ≠ 0°, an anticrossing gap opens up around the intersection between the two modes through mode hybridization. The anticrossing gap is the signature of such hybridization and the gap size between the hybridized modes is defined here as *g*/π which we take at zero field as this is the point where the uncoupled modes would intersect at θ = 45°. Equation 3 can then be used to fit experimental data as described in the following section. Solving eq 3 for θ = 0°, we can obtain the frequency versus field dependence of the bare acoustic and optical modes, BAM and BOM, respectively, given by eq S8 in the Supporting Infomation.

The experimental setup is depicted in Figure 2c. A typical frequency swept spectrum is shown in Figure 2d which can be fitted with two differentiated Lorentzian peaks (see the Supporting Infomation, eq S9), from which the resonance frequency can be extracted. In Figure 2e, we plot a 2D map of the spectra obtained when θ ≈ 90° at 15 K. We observe two modes that can be fitted by BAM and BOM described in eq 3. Furthermore, simulations at θ = 0° shown in Figure S3b in the Supporting Infomation demonstrate the intersection of the two modes along this high-symmetry axis. This observation confirms the absence of a symmetry-breaking condition in these orientations.

In Figure 2f, we plot the 2D map of the spectra obtained at 15 K in the in-plane orientation with θ ≈ 45°. Here, we clearly observe magnon–magnon coupling, as evidenced by the opening of the anticrossing gap, which is comparable to the bare magnon frequencies and hence in the ultrastrong coupling regime. These two hybridized modes can be well fitted with the computation of eq 3.

Two other modes are also observed in the 2D map. The mode that crosses the LFHM at approximately 1.5 T in Figure 2f is identified as a harmonic resonance of the HFHM mode and appears at half the resonant frequency of the HFHM. Additionally, double, quarter, and eighth harmonics are observed, which are fully depicted in Figure S4 of the Supporting Infomation. The other mode, a linear mode reaching 40 GHz at approximately 1.2 T, is explained by background absorption originating from the waveguide itself. Reference measurements are provided to confirm this (see the Supporting Infomation, Figure S5).

In Figure 2g,h, we present micromagnetic simulations carried out in Mumax3, for θ = 90° and θ = 45°, respectively. The simulation is carried out using the magnetic parameters determined from fitting at 15 K and shows good agreement with the experimental data as shown in Figure 2e,f.

By fitting the experimental resonant frequencies for the coupled modes, we can extract *H*_A1_ and *H*_A2_. Furthermore, we calculate the coupling strength as half the gap size, *g*/2π, of the coupled modes at the field at which the two uncoupled modes would intersect (≈ 0 T when θ = 45°).

**Figure 3:**
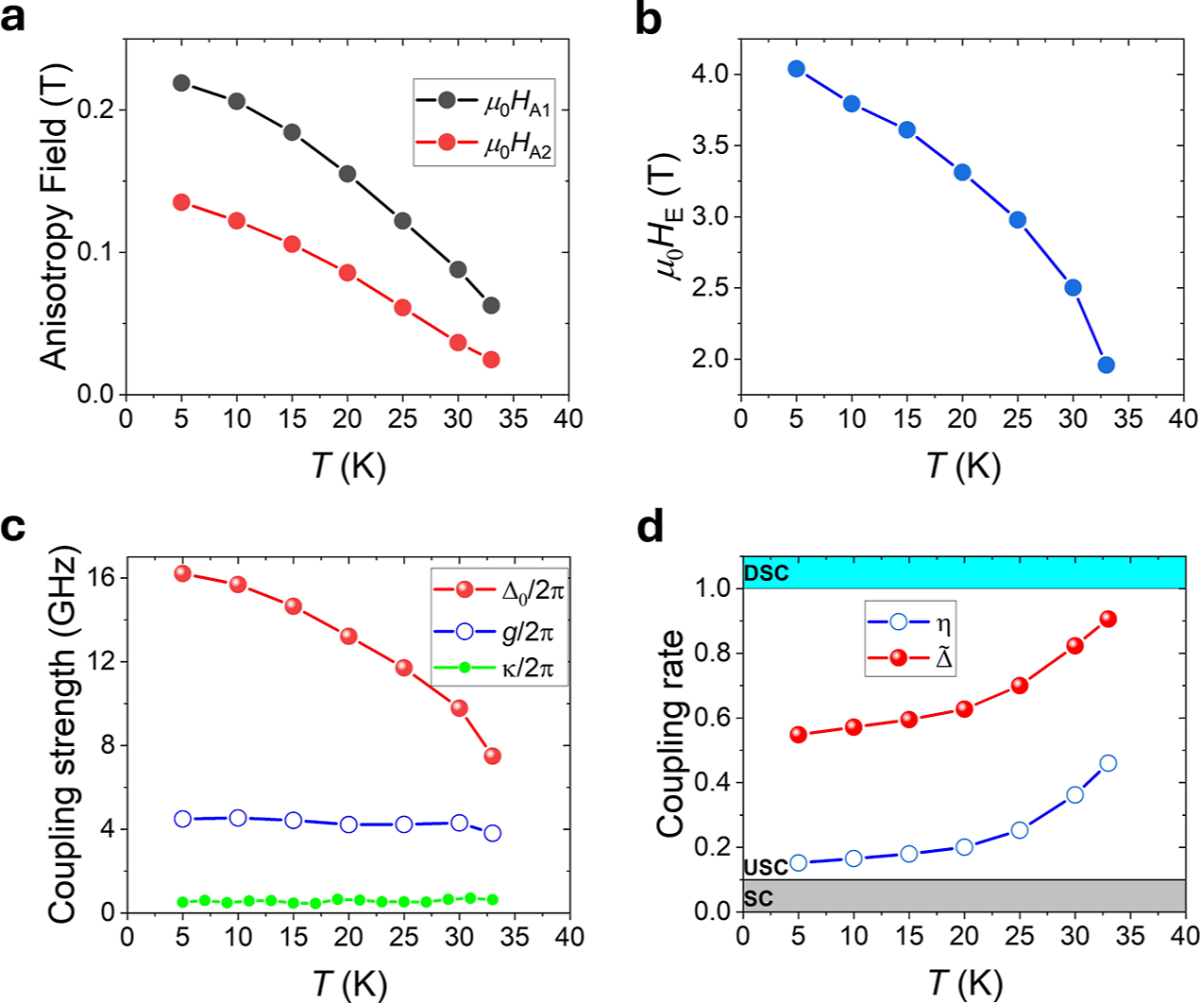
Temperature dependence of magnetic parameters and coupling. (a,b) Plot showing the temperature dependence of μ_0_*H*_A1_, μ_0_*H*_A2_ (a), and μ_0_*H*_E_ (b). (c) Temperature dependence of the intrinsic coupling strength, Δ_0_/2π, coupling strength, *g*/2π, and the line width, κ/2π. A significant increase between the two methods of calculating coupling strength is observed. (d) Temperature dependence of the coupling rate determined through the two methods, gap size η, and intrinsic coupling Δ̃. The intrinsic coupling rate is seen to approach the deep-strong coupling regime with temperature. SC, USC, and DSC represent strong coupling, ultrastrong coupling, and deep-strong coupling regimes, respectively.

The values obtained for *H*_A1_ and *H*_A2_ are presented in Figure 3a. Throughout all temperatures, the value of *H*_A1_ remains larger than *H*_A2_. Both anisotropy fields follow a decreasing function with temperature as is typical of magneto-crystalline anisotropies.^37^ In Figure 3b, we plot the values of *H*_E_ versus *T* which also decreases as a function of temperature as would be expected and agreeing with recent results using magnetic torque.^38^

It is typical to use the coupling rate, η, by comparing the anticrossing gap size to the uncoupled excitation frequency, where η is given by the following
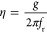
4Here, *f*_r_ represents the bare excitation frequency at the crossing of the uncoupled BAM and BOM; for θ = 45°, this occurs at ≈ 0 T.

Similarly, we can now define the intrinsic coupling rate, Δ̃, as follows from the intrinsic magnon–magnon coupling strength defined in eq 3
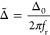
5where Δ_0_ is the value of Δ at the point of intersection of the uncoupled modes.

In Figure 3c, we compare the values of Δ_0_/2π and *g*/2π to the line width, κ/2π, of the hybridized modes. The line width is taken at a field of 200 mT. No clear field or temperature dependence of the line width is observed due to large extrinsic broadening (see the Supporting Infomation, Figure S6) as is common in bulk vdW samples where inhomogeneity across the sample makes line width analysis difficult.^39^ In both Δ_0_/2π and *g*/2π, we show that the coupling strengths are significantly larger than κ/2π meeting the criteria for strong coupling.^19,22^ Furthermore, we observe that Δ_0_/2π is significantly larger than *g*/2π over the entire temperature range. This agrees with the findings of Li et al.^30^ in asymmetric SyAFs, which suggest that the gap size should not be used to quantify the coupling strength in cases of intrinsic symmetry-breaking-induced magnon–magnon coupling.

We now compare the coupling rates as a function of temperature obtained through the two methods, η and Δ̃, as shown in Figure 3d. We observe a significant difference between the two methods at all temperatures, with Δ̃ being more than twice the value of η. Furthermore, Δ̃ increases as a function of *T*, from a minimum of 0.55 at 5 K to a maximum of 0.91 at 33 K, approaching the deep-strong regime as the temperature approaches *T*_N_. This temperature dependence arises from the interplay between the magnetic parameters as a function of temperature, as we demonstrate below.

**Figure 4:**
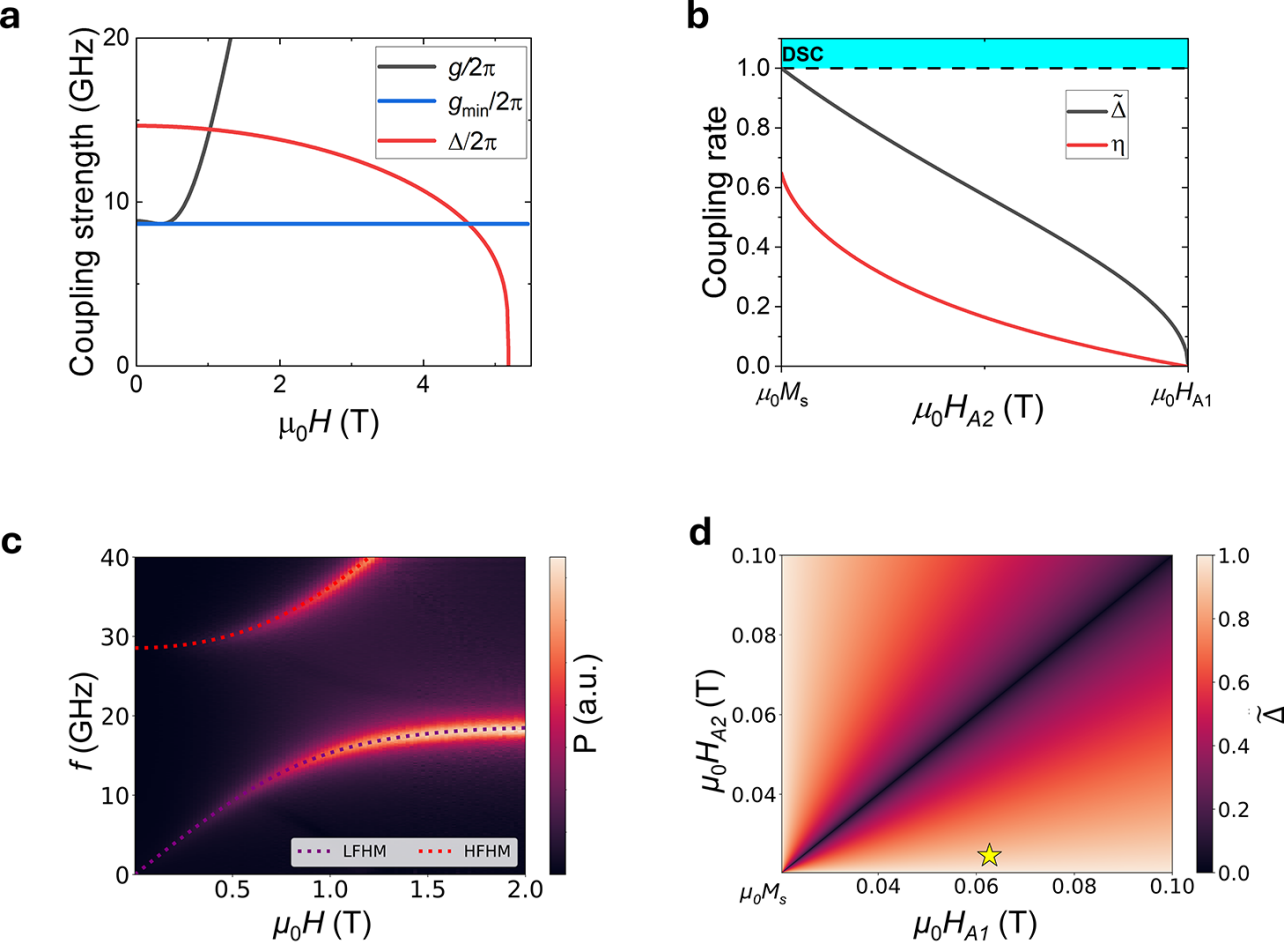
Tunable coupling rate via magnetic anisotropy up to deep-strong coupling. (a) Field dependence of Δ/2π compared to the *g*/2π at 15 K, where *g*_min_/2π is the half of the minimum gap size. (b) Analytical values of η and Δ̃ as a function of *H*_A2_; when μ_0_*H*_A2_ is equal to μ_0_*M*_s_, the deep-strong regime is reached. (c) Micromagnetic simulation of the FMR spectra for the deep-strong coupling case, which shows good agreement with the analytical solutions. (d) 2D plot of Δ for a fixed value of *M*_s_ at 33 K as a function of *H*_A2_ and *H*_A1_, demonstrating the potential tunability of the coupling rate. The star indicates the position and value of Δ̃ observed experimentally at 33 K.

In Figure 4a, we plot the field dependence of Δ/2π compared to *g*/2π as a function of field at an example temperature of 15 K. We demonstrate a notable difference between the two parameters at the intersection field (≈ 0 T when θ = 45°). Additionally, we observe that Δ/2π is field dependent, as it is determined by the canting angle between the sublattice moments. Δ/2π reaches a maximum when the moments are antiparallel at zero field and becomes zero when the canting angle equals 45°. This contrasts with the field-independent Δ/2π introduced for the SyAF, where the intrinsic symmetry breaking arises from differing *M*_s_ values between the layers and has no field dependence.^30^

To further investigate the coupling rate, we compare the simulated coupling rate obtained from both methods as a function of *H*_A2_, as shown in Figure 4b. Here, η is observed to increase as *H*_A2_ decreases, reaching a value of approximately 0.65. At this value, *H*_A2_ becomes less than *M*_s_, and micromagnetic simulations show that the system transitions into an in-plane eas*y*-axis system (see the Supporting Infomation, Figure S7). On the other hand, Δ̃ increases, reaching a maximum value of 1 when *H*_A2_ equals *M*_s_. We define this point as the critical coupling strength, corresponding to the deep-strong regime, similar to observations in anisotropic SyAFs.^18,21,40^

In Figure 4c, we present the micromagnetic simulation of this deep-strong coupling case. In this simulation, the lower hybridized mode is observed to reach 0 GHz at 0 T, indicating the maximum achievable coupling rate. These simulated modes are well fitted by analytical solutions, setting μ_0_*H*_A2_ to μ_0_*M*_s_, which yields a coupling rate of 1.

We now derive the analytical solution for Δ̃ from eqs 3 and S7 in the Supporting Infomation, as the field goes to zero, yielding the following expression
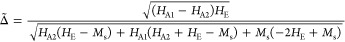
6

Equation 6 provides insight into how to precisely tune the coupling rate through control of the two anisotropy values. In Figure 4d, we present a 2D plot of the Δ̃as a function of μ_0_*H*_A1_ and μ_0_*H*_A2_ for the values of μ_0_*M*_s_ and μ_0_*H*_E_, respectively, at 33 K. This demonstrates the possibility to tune the coupling strength from uncoupled to a critically coupled regime through manipulation of the two anisotropy terms. Avenues for achieving this precise tuning include ionic gating, intercalation, or strain, which have been shown to have considerable effects on anisotropy in vdW magnetic materials.^41−45^

## Conclusions

In conclusion, our investigation has revealed the temperature dependence of the ultrastrong magnon–magnon coupling in the vdW AFM CrPS_4_, approaching the deep-strong regime with a maximum coupling rate of 0.91 achieved at 33 K. This coupling is attributed to the interplay of orthorhombic anisotropies, saturation magnetization, and the exchange field. We evaluated the coupling rate using two approaches: the gap size η and the intrinsic coupling rate Δ̃. Our findings indicate that, in systems with intrinsic symmetry-breaking anisotropy, these two methods yield notably different coupling rates. We further provide an analytical description of Δ̃ in the case of orthorhombic anisotropy, demonstrating how tuning anisotropy values to a critical point could allow for realization of deep-strong coupling. This study not only enhances our understanding of the spin dynamics in vdW AFMs but also demonstrates the potential for vdW AFMs as an ideal system to experimentally reach deep-strong magnon–magnon coupling.

## Methods

### Material Growth and Sample Characterization

Chromium thiophosphate (CrPS_4_) was synthesized in a quartz ampule via physical vapor transport (PVT)—without any transporting agent.^46^ About half of a gram of stochiometric element mixture (metal chromium powder, red phosphorus powder, and elemental sulfur powder (all purchased from Sigma-Aldrich), Cr/P/S = 1:1:4) was ground in an agate mortar, moved into a quartz ampule, evacuated to high vacuum (below 5 × 10^–6^ mbar) by a turbomolecular pump, and closed by a flamer. The sealed ampule was put into a two-zone furnace, that was calibrated in a way that the mixture of elements was kept at 750 °C and the deposition zone was 710 °C. The ampule was warmed from room temperature to designated ones within 5 h, and after 4 days, the furnace was turned off and the sample was allowed to cool down naturally. Then, the ampule was opened, and only recrystallized, large, pure CrPS_4_ crystals from the deposition zone (710 °C) were collected.

The diffractogram of a single, as-grown CrPS_4_ crystal placed in the preferential (001) orientation was recorded using a thin-film X-ray diffractometer (TL-XRD), Bruker D8 Advance, equipped with a Cu anode and a parallel beam.

Energy-dispersive X-ray spectroscopy (EDS) and electron micrographs were obtained using a scanning electron microscope, Zeiss Sigma 300, equipped with an Oxford EDS AztecLive 60 mm^2^ area EDS detector. The experiments were performed at an acceleration voltage of 20 kV without any sample coating.

### Measurement Techniques

To investigate the magnetism and spin dynamics in the vdW AFM CrPS_4_, we performed magnetometry and ferromagnetic resonance (FMR) measurements using a Quantum Design Physical Property Measurement System (PPMS). This PPMS configuration allows for the application of magnetic fields up to 9 T and provides precise temperature control down to 1.8 K. The system is equipped with a vibrating sample magnetometer (VSM) and a NanOsc CryoFMR probe for CPW-FMR spectroscopy, covering a frequency range of 2–40 GHz.

Broadband microwave spectrum measurements were performed at low temperatures using a CPW with a center conductor width of *w* = 2 mm. We conducted field-modulated, frequency-sweep measurements at a constant magnetic field and temperature to generate 2D dispersion maps.

### Micromagnetic Simulation

Dynamic micromagnetic simulations were performed using the Mumax3 package.^47^ CrPS_4_ was modeled on a 5 × 5 × 25 nm^3^ cell grid with dimensions 150 × 250 × 50 nm^3^. The upper and lower cells in the out-of-plane direction were antiferromagnetically coupled with an exchange scaling of −3.04 and exchange stiffness of 10 × 10^–12^ J/m. Gilbert damping was set to 0.01 and the simulation temperature was 0 K. The simulation is carried out using the magnetic parameters determined from magnetometry. FMR spectra are obtained using sinc field pulses in combination with an applied external dc field. The sinc pulse period was set to 10 ns and the time-varying magnetization responses were Fourier transformed to obtain the final spectra.

## Data Availability

All relevant data are available from the corresponding author upon reasonable request.

## Supporting Information Available

Out-of-plane field measurements; θ ≈ 0° in-plane angle measurements; temperature-dependent FMR spectra; harmonic resonance in reference sample; line width as a function of field and temperature; simulation of in-plane anisotropy transition; resonance frequency derivation; Lorentzian fitting equations; simulation of phase difference for acoustic and optical modes; temperature dependence of γ/2π; and EDS spectrum of crystal and image of sample on waveguide with dimensions (PDF)
