# Supplementary material for: Tunable Ultrastrong Magnon–Magnon Coupling Approaching the Deep-Strong Regime in a van der Waals Antiferromagnet
Source: ACS Nano. 2025 Apr 17;19(16):16024–31. doi: 10.1021/acsnano.5c02576 (PMC12045018; doi:10.1021/acsnano.5c02576)
Supplement: Supplementary file 1 — nn5c02576_si_001.pdf [file nn5c02576_si_001.pdf]

Supplementary Information for "Tunable Ultra-Strong Magnon-Magnon Coupling Approaching the Deep-Strong Regime in a van der Waals Antiferromagnet "

Charlie W. F. Freeman[1,2,*], Harry Youel[1], Adam K. Budniak[3,4], Zekun Xue[1], Henry De Libero[5], Thomas Thomson[5], Michel Bosman[4,6], Goki Eda[3,7,8], Hidekazu Kurebayashi[1,9,10] & Murat Cubukcu[1,2,†]

[1] London Centre for Nanotechnology, University College London, London, WC1H 0AH, UK
[2] National Physical Laboratory, Teddington, TW11 0LW, UK
[3] Department of Physics, National University of Singapore, 117551, Singapore
[4] Department of Materials Science & Engineering, National University of Singapore, 117581, Singapore
[5] Department of Computer Science, University of Manchester, Manchester, M13 9PL, UK
[6] Institute for Materials Research and Engineering, Agency for Science, Technology and Research (A*STAR), 138634, Singapore
[7] Department of Chemistry, National University of Singapore, 117543, Singapore
[8] Centre for Advanced 2D Materials, National University of Singapore, 117542, Singapore
[9] Department of Electronic and Electrical Engineering, University College London, London, WC1E 7JE, UK
[10] WPI-AIMR, Tohoku University, 2-1-1, Katahira, Sendai 980-8577, Japan
* uceecwf@ucl.ac.uk † m.cubukcu@ucl.ac.uk

Ultra-strong magnon–magnon coupling in the presence of an out-of-plane magnetic field and monoclinic anisotropy

Measurements in the $H//c$ orientation reveal complex spectra. In the uniaxial AFM case, we expect two linear modes in the AFM phase that are degenerate at zero field [1]. These modes correspond to the left-handed low-frequency (HFM) and right-handed high-frequency (LFM) modes depicted in the diagram in Fig. S1(a-b). Furthermore, in the canted regime for the uniaxial case, one would expect a single Kittel-like acoustic mode, with the out-of-phase mode having a resonance frequency of zero. The first distinction to note in our data shown in Fig. S1(d) is the non-degeneracy at zero-field (δ) and non-linear dependence of the HFM and LFM modes below the spin-flop transition. This observation has been previously reported in other layered materials such as $Mn_2P_2S_6$ and $BaCu_2Si_2O_7$, and has been identified as originating from rhombic or cubic anisotropy terms in the

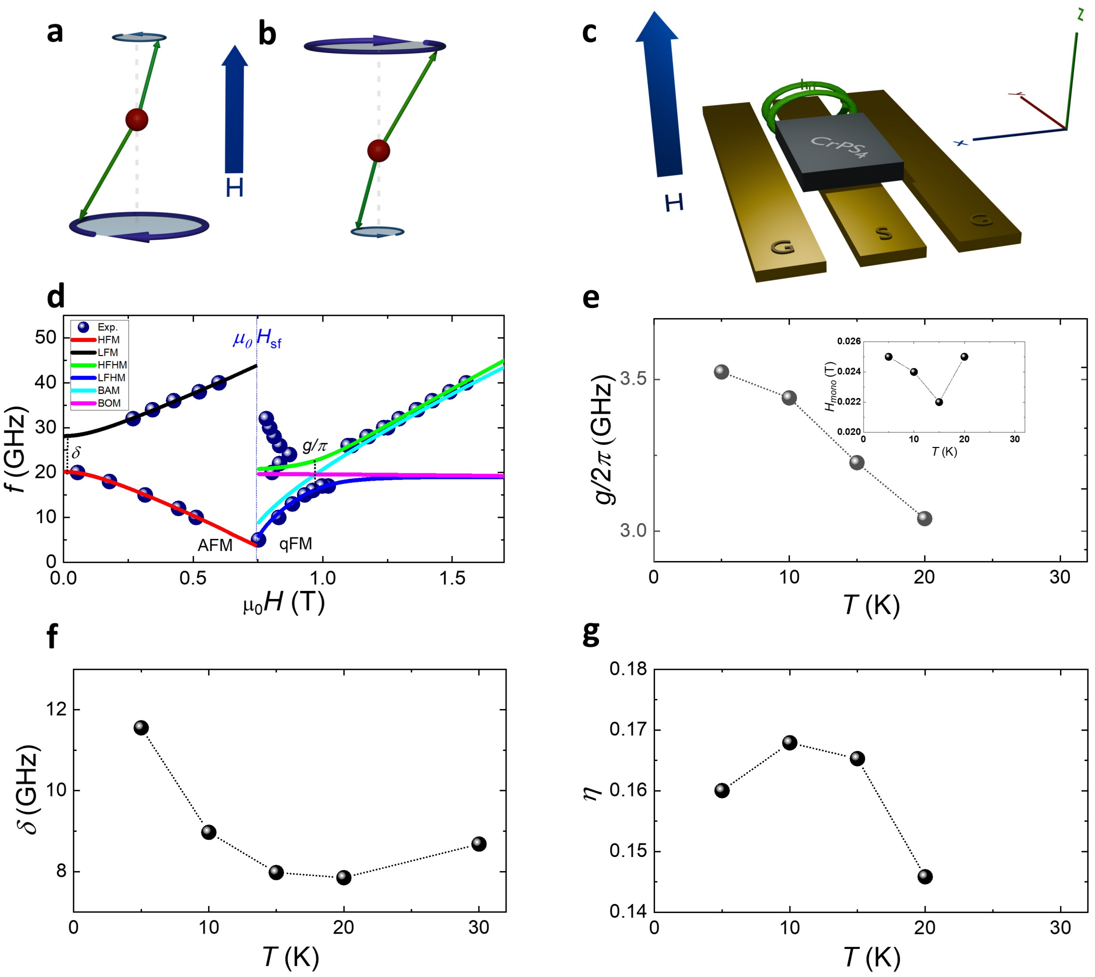

Figure S1: **Spin dynamics in $CrPS_4$ under an out-of-plane magnetic field.** (a-b) Schematic illustration of the magnetic moments' precession processes in the a right-handed high-frequency mode (HFM) (a) and a left-handed low-frequency mode (LFM) (b). (c) Schematic of the configuration between coplanar waveguide (CPW), $CrPS_4$ crystal, and external out-of-plane magnetic field (H). (d) Frequency (f) dependent ferromagnetic resonance results obtained at 15 K, showing the magnetic field applied (μ_0H) along the z-axis (dots) and the fitting including monoclinic anisotropy. (e) The coupling gap size ($g/2\pi$) as a function of temperature. The inset shows the monoclinic anisotropy field (H_{mono}) as a function of temperature. (f) The non-degenerate gap at zero magnetic field (δ) as a function of temperature. (g) The normalised magnon-magnon coupling strength (η) as a function of temperature.

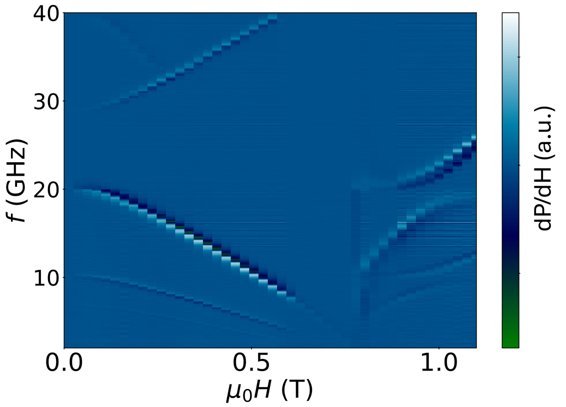

Figure S2: **Out-of plane 2D map at 15 K**. Frequency swept spectra of the out-of plane modes plotted as a 2D map.

free energy equation [2, 3], which agrees with the findings of the section above where we employ an orthorhombic anisotropy. Above the H_{sf}, a clear signature of magnon-magnon coupling is observed with the anti-crossing gap of the two modes. This differs from the expected spectra for a material with orthorhombic anisotropy, where the optical and acoustic modes will not hybridise as the symmetry condition is not broken for an out of plane field. The observation of two distinct modes that couple in the canted regime implies an intrinsic symmetry-breaking phenomenon must be present. It has been proposed that by considering the monoclinic nature of the crystal lattice, it is plausible to introduce a monoclinic anisotropy term, K_{mono}, into the free energy equation given in Eq. 1 (main manuscript) [4]:

$$\begin{aligned} F_{\mathrm{A}} =& K_1/2(\alpha_1^2+\beta_1^2)+K_2/2(\alpha_2^2+\beta_2^2) \\ &+K_{\mathrm{mono}}(\alpha_1\alpha_3+\beta_1\beta_3) \end{aligned} \tag{S1}$$

By including this term, we can follow the same method as detailed above and fit the obtained spectra, confirming the validity of the monoclinic anisotropy. We find the values of this anisotropy term to be an order of magnitude lower that the orthorhombic terms which agrees with the findings from Li et al. [4]. We present values of the monoclinic anisotropy value over a range of temperatures between 5 K and 20 K (Fig. S1(e) inset). In Fig. S1(e), we show the obtained half gap size $g/2\pi$ for T where a clear anticrossing gap could be observed. We observe a decreasing trend for the range between 5 K and 20 K with a maximum value of 3.5 GHz at 5 K. For temperatures above 20 K, no magnon-magnon coupling signatures were investigated as the data collected was difficult to measure. However, a more detailed study of the unusual monoclinic symmetry-breaking anisotropy could be carried out in future studies. In Fig. S1(d), we observe the spectra sharply decreasing in frequency as H around H_{sf}. This phenomenon, known as orientation resonance has been observed previously in $Mn_2P_2S_6$ [2] and is a consequence of the spin flop transition. In Fig. S1(f), δ is shown as a function of temperature, we show that it is decreasing as a function of temperature. In Fig. S1(g), we show η for a range of temperatures, we find a maximum value of 0.17 at 10 K. This is smaller than that found for the in-plane regime as we are not at the maximum coupling angle when the applied field is directly out of plane. Maximum coupling is observed when the field is tilted by 12.5° [4].

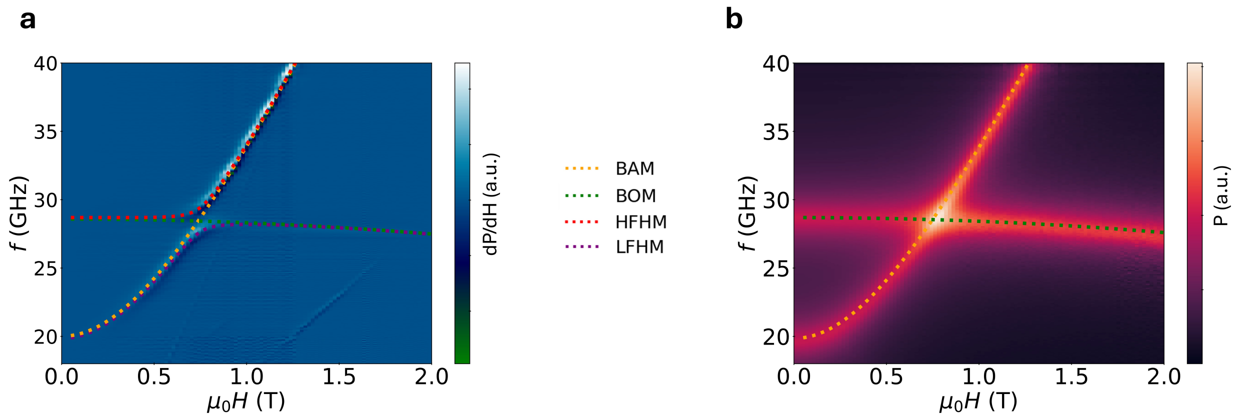

Figure S3: **In-plane FMR spectra at $\theta \approx 0^{\circ}$** (a) Experimental 2D spectra at small angle (approximately 7°) taken at 15 K. A small gap opening is observed as the angle is not exactly 0°. (b) Simulated spectra at $\theta = 0^{\circ}$, no gap opening is observed due to the symmetry conservation in this condition. BAM and BOM modes from the experimental data are plotted and show good agreement with the simulation data.

Ultra-strong magnon–magnon coupling with an in-plane magnetic field at $\theta \approx 0^{\circ}$

Due to the sensitivity of any deviations away from $\theta = 0°$, it is difficult to experimentally show the crossing of the two modes at $\theta = 0°$. We present in Fig. S3(a) a 2D map at small angle. Fitting this results in a deviation of approximately $\theta \approx 7^{\circ}$, this result shows the decrease of the size of the gap. Micromagnetic simulations presented in Fig. S3(b) were carried out for $\theta = 0°$. In this case, the modes are observed to cross with no interaction due to the symmetry conservation in this configuration. BAM and BOM modes calculated from fitting of Fig. 2(f) of the main manuscript are plotted and fit well with the simulated spectra.

Temperature-dependent ultra-strong magnon–magnon coupling with an in-plane magnetic field at $\theta \approx 45^{\circ}$

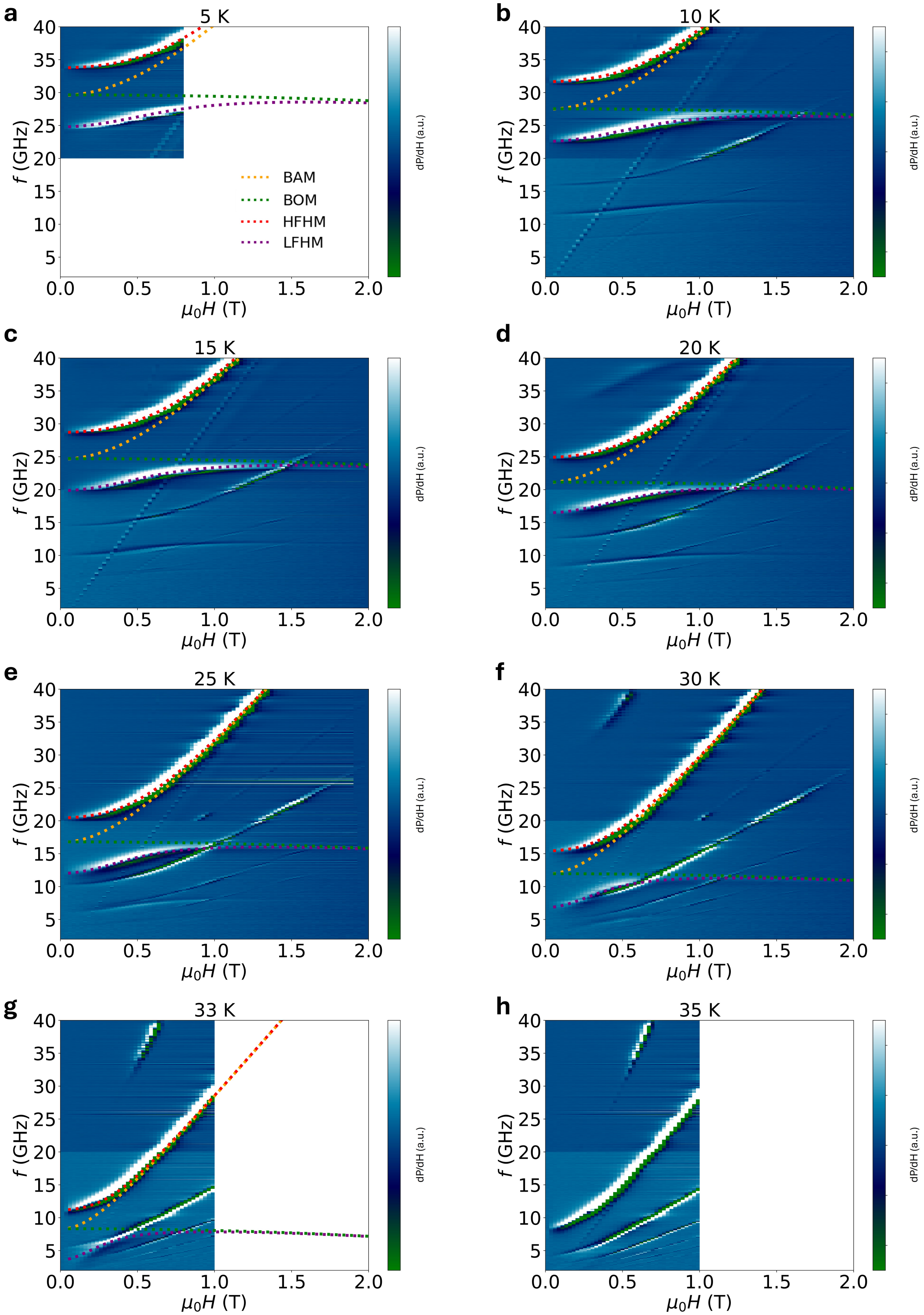

Figure S4: **Temperature dependence FMR spectra**. (a-h) 2D maps of the FMR spectra obtained for all temperatures. The fitted modes are plotted and with the legend given in (a). For all temperature the harmonic modes are also visible.

Harmonic resonances and background absorption

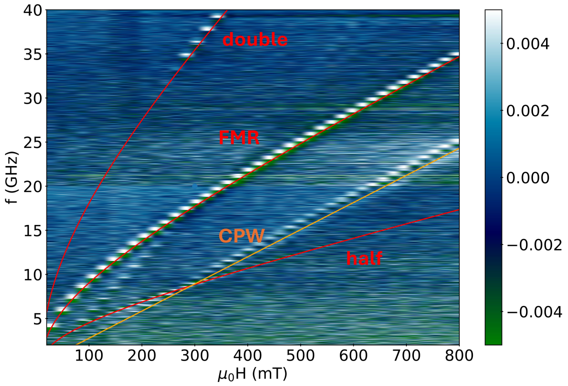

Figure S5: **Permalloy reference with harmonic modes.** 2D map of the FMR spectra for a permalloy (10nm) reference sample. The harmonic modes (red) are observed with reduced intensity and the background CPW mode (orange) is also shown.

In Fig. S5, we show in-plane magnetic field frequency swept measurements at 15 K on a reference permalloy (10nm) sample, in order to replicate the harmonic modes observed in the $CrPS_4$ sample. While the waveguide may produce small harmonic resonances it is interesting that the intensity of these harmonic resonances are much greater than that observed in the reference thin film permalloy sample. Whether this strong harmonic absorption is a consequence of the vdW nature of the crystal or the increased absorption due to the large volume of the bulk crystal of the crystal may be interesting for future investigations.

Linewidth of the hybridised modes

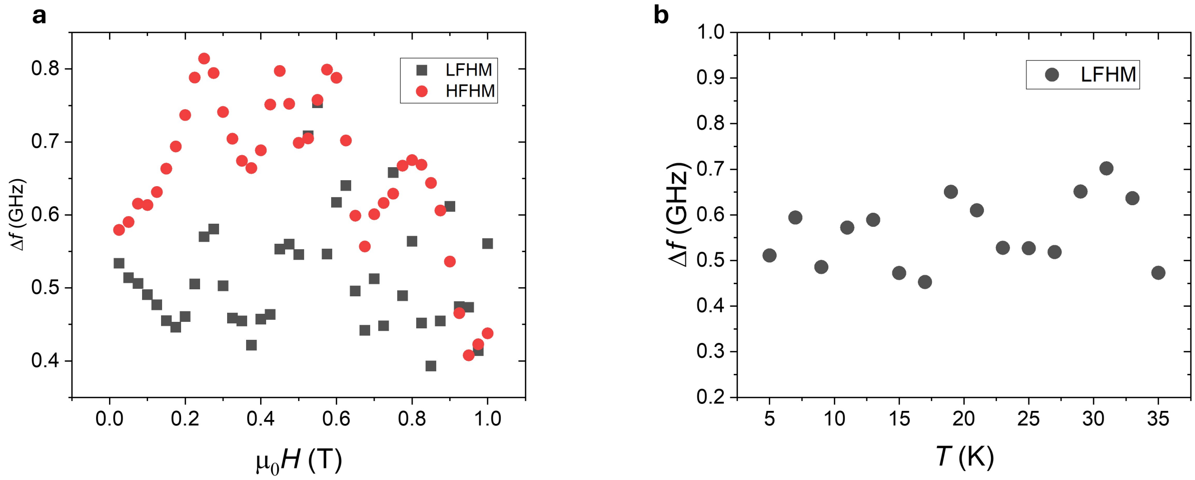

Figure S6: **Linewidth as a function of field (a) and temperature (b).** No clear dependence is observed in either due to inhomogeneity in the bulk sample.

Plotting the linewidths of the coupled modes versus field (a) and temperature (b) in Fig. S6, we are not able to observe any clear dependence. This is most likely due to the poor fitting

of linewidth due to multi peak behavior at resonance due to inhomogeneity that is unavoidable in bulk vdW crystals. In order to extract damping parameters for this material, thin exfoliated samples would need to be studied.

In-plane easy axis transition

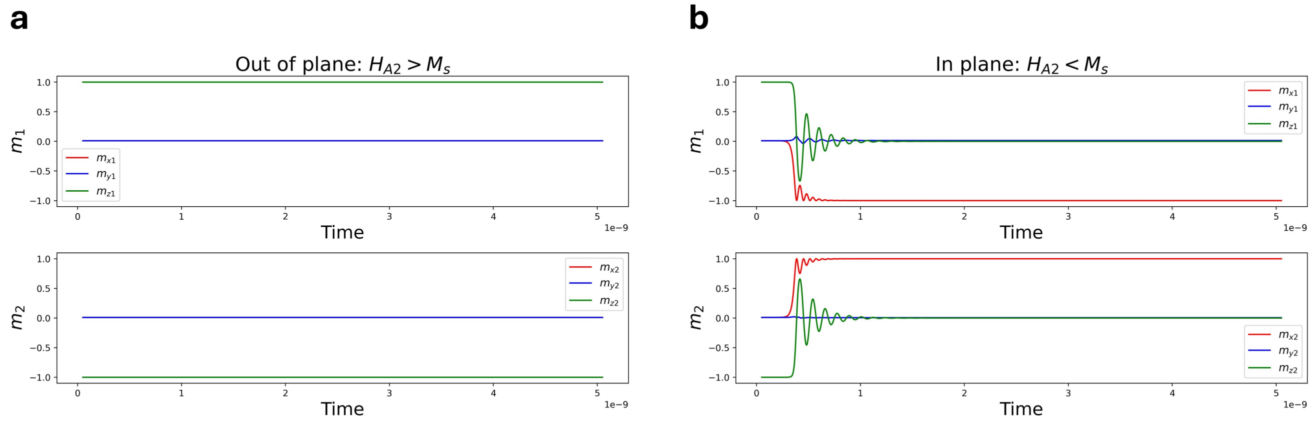

Figure S7: **Transition to an in-plane easy axis when $H_{A2} < M_s$.** Micromagnetic simulations of the components of magnetisation in x, y and z of the two sublattices, in two conditions (a) $H_{A2} > M_s$ and (b) $H_{A2} < M_s$. We observe that when $H_{A2} < M_s$ the system relaxes to in-plane under a small applied field, indicating a switching of the easy-axis from out-of plane to in-plane.

Micromagnetic simulations shown in Fig. S7(a-b) show relaxation in time under a small in-plane applied field for two values of H_{A2}, (a) $H_{A2} > M_s$ and (b) $H_{A2} < M_s$. The system is initialised with the sublattices out-of plane in order to exclude metastable configurations. We observe that when $H_{A2} < M_s$ the system relaxes to in-plane under a small applied field, indicating a switching of the easy-axis from out-of plane to in-plane. Simulations of the FMR spectra in this setup were unable to be produced due to different relaxation times interfering with the FFT. However, this is similar to the case of SyAF studied by Wang et al. [4]. In this case, when the anisotropy switches to the in-plane direction the coupling strength is seen to decrease from the maximum value. We expect the same trend in our system.

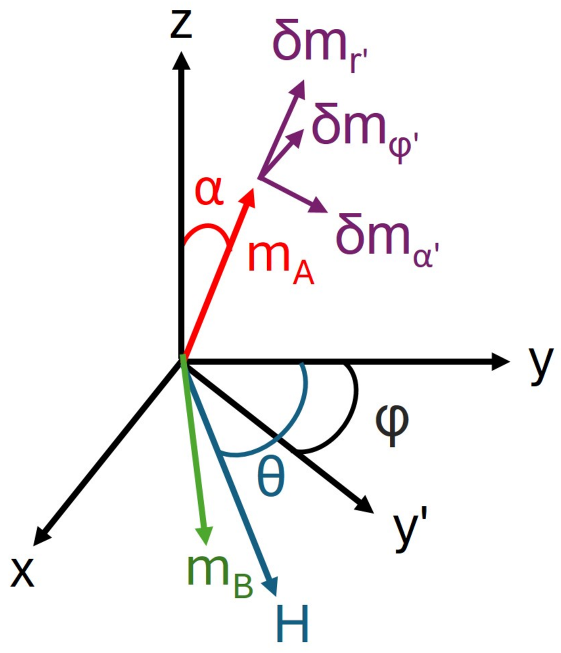

Figure S8: **Coordinate system for in-plane field equilibrium equation.**

Theory

To calculate the resonance frequencies as a function of field we follow the method set out by Li et al. [5] which we include for completeness here. The free energy equation for orthorhombic anisotropy is proposed by Keffer et al. [6] where we set K_3 and K_4 to zero. The coordinate system adopted is displayed in Fig. S8 for H in plane with an angle θ from the y axis, where x, y, z corresponding to the a, b and c axis respectively. A rotation of coordinates into $\delta m_{r'}$, $\delta m_{\alpha'}$ and $\delta m_{\alpha'}$ is defined with respect to $\hat{m}_A$ Next the equilibrium equation is defined such that $\hat{m}_A$ aligns with the total effective field. The equilibrium equations are given by the following,

$$\begin{aligned} H\cos(\varphi-\theta) &= \left(2H_E - M_s + H_{A1}\sin^2\varphi + H_{A2}\cos^2\varphi\right)\sin\alpha \\ H\sin(\varphi-\theta) &= (H_{A2}-H_{A1})\sin\alpha\sin\varphi\cos\varphi \end{aligned} \tag{S2}$$

The interlayer exchange energy is given by $\mu_0 M_s H_E \hat{m}_A \cdot \hat{m}_B$. We do not include biquadratic terms in this approximation. This is equation is solved numerically to find α and φ for values of H, the values are of the same value for $\hat{m}_B$. Next the LLG equation is given for the two sublattices as defined in the main manuscript Eq. 2. This is then expanded with $\hat{m}_i = \hat{m}_i^e q + \delta m_i e^{i\omega t}$ $(i = A, B)$. where the latter term is the precession of the magnetic moments about the equilibrium position, resulting in

$$\begin{aligned} i\omega\delta m_i =& \mu_0\gamma\hat{m}_i^e q \times (H_E - M_s\delta m_i + H_E\delta m_j + M_s(\delta m_i\cdot\hat{z})\hat{z} + \\ & H_{A1}(\delta m_i\cdot\hat{x})\hat{x} + H_{A2}(\delta m_i\cdot\hat{y})\hat{y} i, j = A, B \end{aligned} \tag{S3}$$

Two new vectors are defined, namely

$$\begin{aligned} \delta m_+ &= \delta m_1 + C_{2y'}\delta m_2, \\ \delta m_- &= \delta m_1 - C_{2y'}\delta m_2, \end{aligned} \tag{S4}$$

where $C_{2y'}$ corresponds to a 180 degree rotation about the y' axis. Substituting into Eq. S4,

$$\begin{aligned} i\omega\delta m_{\pm} =& \mu_0\gamma\widehat{m}_1^{eq} \times \left(H^{eq}\delta m_{\pm} \pm H_E C_{2y}\delta m_{\pm} + M_S\left(\delta m_{\pm}\cdot\hat{z}\right)\hat{z}\right. \\ &+ \frac{H_{A1}}{2}\delta m_{\pm}\left(\cdot\hat{x}\hat{x} + \left(\cdot C_{2y,}\hat{x}\right)\left(C_{2y,}\hat{x}\right)\right) + \frac{H_{A1}}{2}\delta m_{\mp}\left(\cdot\hat{x}\hat{x} - \left(\cdot C_{2y,}\hat{x}\right)\left(C_{2y,}\hat{x}\right)\right) \\ &+ \frac{H_{A2}}{2}\delta m_{\pm}\left(\cdot\hat{y}\hat{y} + \left(\cdot C_{2y},\hat{y}\right)\left(C_{2y},\hat{y}\right)\right) + \frac{H_{A2}}{2}\delta m_{\mp}\left(\cdot\hat{y}\hat{y} - \left(\cdot C_{2y},\hat{y}\right)\left(C_{2y},\hat{y}\right)\right) \end{aligned} \tag{S5}$$

This can be represented as a matrix equation by the following,

$$iw\begin{pmatrix}\delta m_{+,\alpha\prime}\\ \delta m_{+,\varphi\prime}\\ \delta m_{-,\alpha\prime}\\ \delta m_{-,\varphi'}\end{pmatrix} = \mu_0\gamma\begin{pmatrix}0 & H_{o12} & H_{o13} & 0\\ H_{o21} & 0 & 0 & H_{o24}\\ H_{o31} & 0 & 0 & H_{o34}\\ 0 & H_{o42} & H_{o43} & 0\end{pmatrix}\begin{pmatrix}\delta m_{+,\alpha\prime}\\ \delta m_{+,\varphi\prime}\\ \delta m_{-,\alpha\prime}\\ \delta m_{-,\varphi}\end{pmatrix} \tag{S6}$$

where,

$$\begin{aligned} H_{o12} &= M_s - H_{A1}\cos^2\varphi - H_{A2}\sin^2\varphi, \\ H_{o13} &= \left(H_{A1} - H_{A2}\right)\cos\alpha\sin\varphi\cos\varphi, \\ H_{o21} &= \left(2H_E - M_S\right)\cos^2\alpha + H_{A1}\cos^2\alpha\sin^2\varphi + H_{A2}\cos^2\alpha\cos^2\varphi, \\ H_{o24} &= \left(-H_{A1} + H_{A2}\right)\cos\alpha\sin\varphi\cos\varphi, \\ H_{o31} &= \left(H_{A1} - H_{A2}\right)\cos\alpha\sin\varphi\cos\varphi, \\ H_{o34} &= -\left(\left(2H_E - M_S\right) + H_{A1}\cos^2\varphi + H_{A2}\sin^2\varphi\right), \\ H_{o42} &= \left(-H_{A1} + H_{A2}\right)\cos\alpha\sin\varphi\cos\varphi, \\ H_{o43} &= 2H_E\sin^2\alpha - M_S\cos^2\alpha + H_{A1}\cos^2\alpha\sin^2\varphi + H_{A2}\cos^2\alpha\cos^2\varphi. \end{aligned} \tag{S7}$$

Solutions to this are given through simple computation of the real part of the eigenvalues of $\boldsymbol{A} = i\mu_0\gamma\boldsymbol{H}_o$ the resonance frequency of the two modes for H can then be computed and is used to fit to the experimental resonance spectra. The equations for the BAM and BOM are given by the following,

$$\begin{aligned} \omega_{\mathrm{BOM}} &= \mu_0\gamma\sqrt{\left(-M_\mathrm{s} + H_{A2}\right)\left(2H_\mathrm{E} - M_\mathrm{s} + H_{A1} - \frac{H^2}{2H_\mathrm{E} - M_\mathrm{s} + H_{A1}}\right)} \\ \omega_{\mathrm{BAM}} &= \mu_0\gamma\sqrt{\left(2H_\mathrm{E} - M_\mathrm{s} + H_\mathrm{A1}\right)\left[\frac{H^2\left(2H_\mathrm{E} + M_\mathrm{s} - H_\mathrm{A1}\right)}{\left(2H_\mathrm{E} - M_\mathrm{s} + H_\mathrm{A1}\right)^2} - M_\mathrm{s} + H_\mathrm{A1}\right]} \end{aligned} \tag{S8}$$

The derivation for the matrix equation for an out of plane field follows the same derivation with the addition of a monoclinic anisotropy term and is detailed fully in [5].

Lorentzian Fit

FMR spectra fitting shown in Fig. 2(d) in the main manuscript is carried out with a two peak derivated Lorentzian given by the following:

$$\begin{aligned} \frac{dP}{dH}(f) = A_1\cdot\left(\frac{\Delta f_1\cdot\left(\Delta f_1^2 - \left(f - f_r^1\right)^2\right)}{\left(\Delta f_1^2 + \left(f - f_r^1\right)^2\right)^2}\right) - S_1\cdot\left(\frac{2\Delta f_1^2\cdot\left(f - f_r^1\right)}{\left(\Delta f_1^2 + \left(f - f_r^1\right)^2\right)^2}\right) \\ + A_2\cdot\left(\frac{\Delta f_2\cdot\left(\Delta f_2^2 - \left(f - f_r^2\right)^2\right)}{\left(\Delta f_2^2 + \left(f - f_r^2\right)^2\right)^2}\right) - S_2\cdot\left(\frac{2\Delta f_2^2\cdot\left(f - f_r^2\right)}{\left(\Delta f_2^2 + \left(f - f_r^2\right)^2\right)^2}\right) + c\cdot f + d \end{aligned} \tag{S9}$$

where $\frac{dP}{dH}$ is the measured transmitted power derivative, Δf is the half width half maximum and f_r is the resonant frequency, A and S and fitting constants for the asymmetric an symmetric components, respectively, c and d account for the slope or constant offset of the measured spectra, respectively. Subscripts 1 or 2 denote the first and second peak.

Phase difference

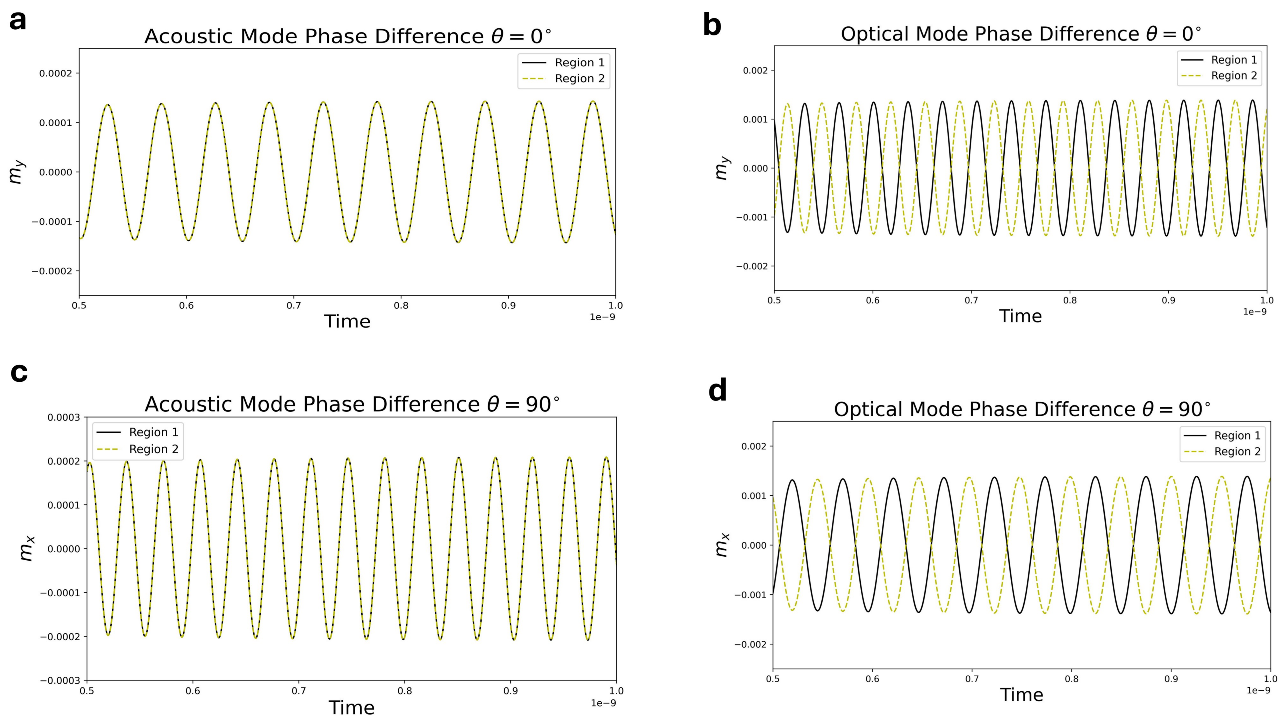

Figure S9: **Phase-resolved analysis of the modes**. Magnetisation components (perpendicular to the applied field) for both regions, corresponding to the acoustic (a–b) and optical (c–d) modes, respectively, for the two angles $\theta = 0°$ and $\theta = 90°$ at 100 mT.

Figures S9(a–b) and S9(c–d) show the magnetisation components for both regions, corresponding to the acoustic and optical modes, respectively, for the two angles with $\theta = 0^\circ$ and $\theta = 90^\circ$. Micromagnetic simulations were conducted to examine the phase difference between the antiferromagnetically coupled regions for both the acoustic and optical modes. These simulations clearly show that the oscillations in the acoustic mode are in-phase, while those in the optical mode are out-of-phase.

Temperature dependence of $\gamma/2\pi$

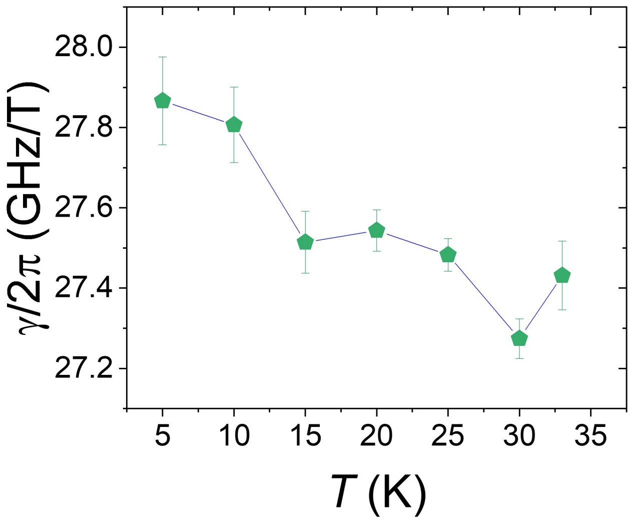

Figure S10: **Temperature dependence of** $\gamma/2\pi$. Plot of $\gamma/2\pi$ as a function of T from the fitting of the hybridised modes. A decreasing trend is observed with T.

Structural Characterisation

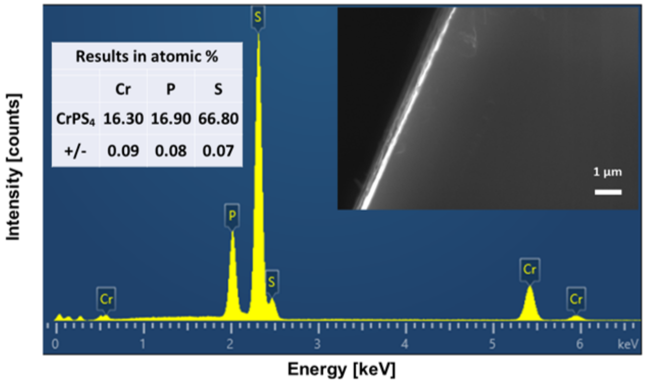

Figure S11: **Structural characterisation.** The EDS spectrum of bulk $CrPS_4$ registered with scanning electron microscope (SEM). All three elements: chromium, phosphorus, and sulfur are present in the sample. The inset shows an InLens micrograph of the crystals, where the series of spectra was recorded. The table presents the atomic composition, based on a few measurements. The Cr:P:S ratio is close to 1:1:4 further confirming receiving $CrPS_4$.

$CrPS_4$ on waveguide

In Fig. S12, we show an image of the $CrPS_4$ crystal placed on the CPW waveguide. The thickness of the crystal is 0.28 mm .

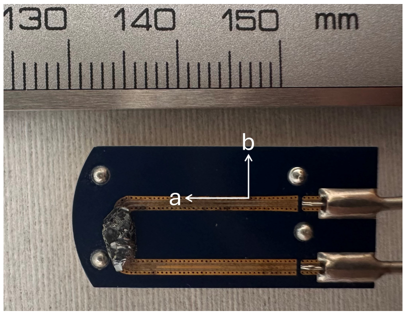

Figure S12: **Image of the $CrPS_4$ crystal and the coplanar waveguide (CPW).**

References

[1] Li, J. *et al.* Spin current from sub-terahertz-generated antiferromagnetic magnons. *Nature* **578** (2019).

[2] Kobets, M. *et al.* Antiferromagnetic resonance in $Mn_2P_2S_6$. *Low Temp. Phys.* **35**, 930–934 (2009).

[3] Hayn, R., Pashchenko, V., Stepanov, A., Masuda, T. & Uchinokura, K. Magnetic anisotropy of $BaCu_2Si_2O_7$: Theory and antiferromagnetic resonance. *Phys. Rev. B* **66**, 184414 (2002).

[4] Li, W. *et al.* Ultrastrong magnon–magnon coupling and chirality switching in antiferromagnet $CrPS_4$. *Adv. Funct. Mater.* **33**, 2303781 (2023).

[5] Li, W. *et al.* Ultrastrong magnon–magnon coupling and chirality switching in antiferromagnet $CrPS_4$. *Adv. Func. Mat.* **33**, 2303781 (2023).

[6] Keffer, F. & Kittel, C. Theory of antiferromagnetic resonance. *Phys. Rev.* **85**, 329 (1952).
